# *De novo* AML exhibits greater microenvironment dysregulation compared to AML with myelodysplasia-related changes

**DOI:** 10.1038/srep40707

**Published:** 2017-01-13

**Authors:** Matheus Rodrigues Lopes, João Kleber Novais Pereira, Paula de Melo Campos, João Agostinho Machado-Neto, Fabiola Traina, Sara T. Olalla Saad, Patricia Favaro

**Affiliations:** 1 Hematology and Transfusion Medicine Center - University of Campinas/Hemocentro - Unicamp, Instituto Nacional de Ciência e Tecnologia do Sangue, Campinas, São Paulo, Brazil; 2 Department of Internal Medicine, University of São Paulo at Ribeirão Preto Medical School, Ribeirão Preto, São Paulo, Brazil; 3 Department of Biological Sciences, Federal University of São Paulo, Diadema, São Paulo, Brazil

## Abstract

The interaction between the bone marrow microenvironment and malignant hematopoietic cells can result in the protection of leukemia cells from chemotherapy in both myelodysplastic syndromes (MDS) and acute myeloid leukemia (AML). We, herein, characterized the changes in cytokine expression and the function of mesenchymal stromal cells (MSC) in patients with MDS, AML with myelodysplasia-related changes (MRC), a well-recognized clinical subtype of secondary AML, and *de novo* AML. We observed a significant inhibitory effect of MDS-MSC on T lymphocyte proliferation and no significant differences in any of the cytokines tested. AML-MSC inhibited T-cell proliferation only at a very low MSC/T cell ratio. When compared to the control, AML-MRCderived MSC presented a significant increase in *IL6* expression, whereas *de novo* AML MSC presented a significant increase in the expression levels of *VEGFA, CXCL12, RPGE2, IDO, IL1β, IL6* and *IL32*, followed by a decrease in *IL10* expression. Furthermore, data indicate that IL-32 regulates stromal cell proliferation, has a chemotactic potential and participates in stromal cell crosstalk with leukemia cells, which could result in chemoresistance. Our results suggest that the differences between AML-MRC and *de novo* AML also extend into the leukemic stem cell niche and that IL-32 can participate in the regulation of the bone marrow cytokine milieu.

Myelodysplastic syndromes (MDS) are heterogeneous clonal haematopoietic stem cell (HSC) disorders that incur an increased risk of evolution to acute myeloid leukemia (AML)^1^, a well-recognized clinical subtype of secondary AML with myelodysplasia-related changes (AML-MRC)^2^. The biological and prognostic differences between *de novo* and secondary AML have been extensively documented, such as the worse outcome of younger patients with secondary AML, compared with *de novo* AML^3^.

HSC self-renewal, differentiation and proliferation are regulated in local tissue microenvironments called niches. One of the main cellular components of the HSC niche are the mesenchymal stromal cells (MSC), which are important regulators of haematopoiesis, as well as of the immune system^4^,^5^. It is rational to assume that MSC, derived from patients with hematological malignancies, harbor some partial defects, either primary or secondary, due to their exposure to altered marrow components. Extensive data have already shown interactions between leukemic cells and their microenvironment, supporting the idea that defects in the HSC microenvironment may play a role either in MDS or in AML development^6^,^7^,^8^,^9^. For instance, interactions between MSC from the leukemic stem cell niche and malignant cells are critical components of resistance to many chemotherapy agents^10^,^11^,^12^.

One of the hallmarks of malignancy^13^, inflammation, has been recognized as an important factor in the pathogenesis of MDS and AML, and involves different molecular and cellular signaling pathways^1^,^14^,^15^,^16^. Thus, the continuous inflammatory state provided by the HSC leukemic niche can contribute to the initiation and progression of diseases. Interleukin (IL)-32 is a proinflammatory cytokine, expressed as several isoforms^17^,^18^, that is thought to contribute to the pathogenesis of infection^19^,^20^,^21^, autoimmune diseases^21^ and cancer^22^,^23^. IL-32 induces inflammatory cytokines such as TNF-α, IL-1β, IL-6, and chemokines through the NF-κB and p38 MAPK signaling pathways^17^. Previous data support a role for IL-32 in the pathophysiology of clonal myeloid diseases^24^.

In this study, we characterized cytokine expression changes and the function of MSC from patients with MDS, AML-MRC and *de novo* AML, in comparison to healthy control (HC) MSC. Moreover, we studied the ability of IL-32 to promote cell proliferation, chemotaxis of leukocytes and chemoprotection towards cytarabine (AraC) in the microenvironment.

## Results

### Expansion and characterization of MSC

MSC were cultured to confluence until the fourth passage. All 8 samples obtained from HC were successfully cultured, while only 71% of the samples obtained from MDS (22 of 31), 70% from AML-MRC (7 of 10) and 71% from *de novo* AML (12 of 17) were able to proliferate. The mean time to reach 80% confluency of samples obtained from MDS and AML-MRC were similar to those of HC (15 ± 6.2; 12.6 ± 6.1; 13.5 ± 2.4 days, respectively, *p* > 0.05).

*De novo* AML cells reached 80% confluency in 21.2 ± 8.2 days, which represents a significantly slower growth than that of HC and AML-MRC samples (*p* < 0.05). Confirming mesenchymal origin, all patient-derived MSC presented a typical profile, standardized by the International Society for Cellular Therapy^25^, and very similar to that of HC-MSC (Supplementary Table 2). Low levels of CD34 and CD31 were sometimes detected in the AML-MRC group, either due to leukemia cell or endothelial cell contamination^26^ or due to macrophage contamination^27^.

To assess the capacity and efficiency for self-renewal of the MSC, a colony-forming unit-fibroblast (CFU-F) assay was performed in some of our patient-derived cells (5 samples of MDS and 2 samples of AML). The MSC derived from MDS patients showed a median of 23.2 CFU-F (range: 16–37) per 10^3^ cells. The samples from MSC-derived AML presented 28 and 8 CFU-F per 10^3^ cells. These results indicate the self-renewal capacity of our MSC derived from patient bone marrow.

### Immunomodulative capability

**Figure 1:**
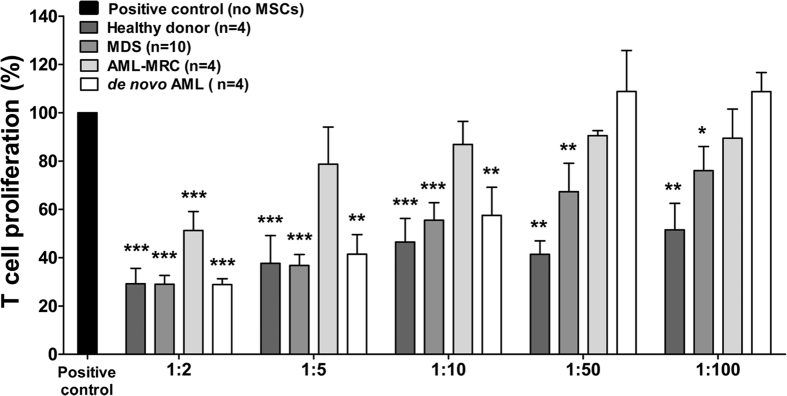
Proliferation of CD3^+^ T cells in coculture with MSC. T cell proliferation assays were performed using CFSE-labeled CD3^+^ T cells activated with PHA and cocultured with MSC (white and shades of gray columns) from healthy donors, myelodysplastic syndromes (MDS), acute myeloid leukemia with myelodysplasia-related changes (AML-MRC) and *de novo* acute myeloid leukemia (*de novo* AML) patients, or without MSC (positive control; black column) for 4 days at MSC:T cell ratios of 1:2, 1:5, 1:10, 1:50 and 1:100 as shown in the figure. Cell proliferation was determined by flow cytometry after gating the lymphocyte population on the forward and side scatter plot and measuring the percentage of CFSE positive T cells. Results are shown as mean ± SEM and the number of samples in each group is shown in the figure. ANOVA, Bonferroni’s post-tests; *p < 0.05, **p < 0.01, ***p < 0.001.

The immunomodulatory properties of MSC have been well characterized^28^, including suppression of T cell-mediated immunity^29^. Herein, the ability of patient-derived MSC to inhibit T cell proliferation was analyzed by mixing different ratios of MSC and CFSE-labeled CD3^+^ cells, in the presence of PHA. We observed a significant inhibitory effect of MDS-MSC on CD3^+^ cell proliferation up to the ratio of 1:100 (*p *< 0.05), in a dose-dependent manner, similar to that of HC-MSC (Fig. 1). This result remained significant when MDS patients were classified into subgroups, according to the WHO 2008 classification (data not shown). On the other hand, in contrast to HC-MSC, which presented normal inhibitory properties, AML-derived MSC were able to significantly inhibit T-cell proliferation at lower ratios. AML-MRC derived-MSC only inhibited proliferation at a ratio of 1:2 (*p *< 0.001), and the *de novo* AML MSC inhibited up to a ratio of 1:10 (*p *< 0.01) (Fig. 1). Further studies are needed to address the key aspects of the reduction of AML-MSC-mediated immunosuppression.

### Cytokine profile in MSC

**Figure 2:**
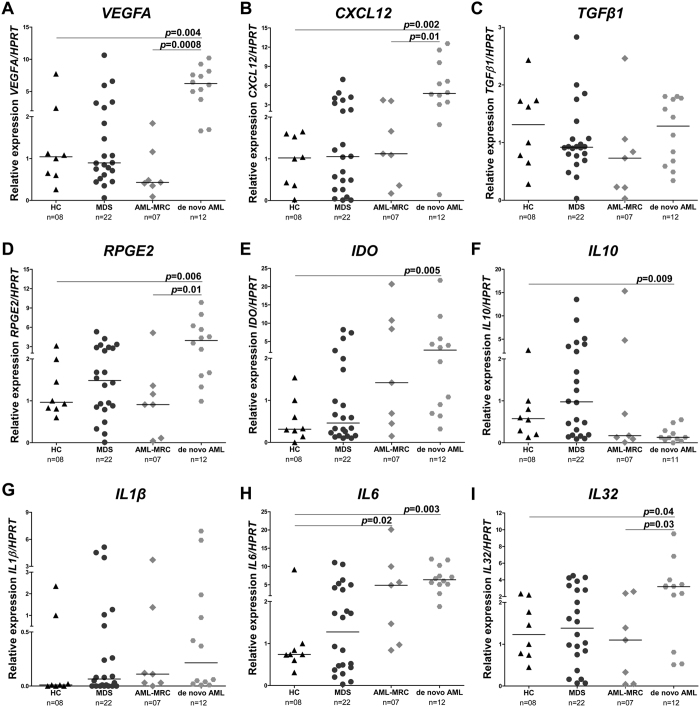
Modulation of cytokines and molecules is mostly observed in the *de novo* AML group. qPCR analyses of mRNA expression of *VEGFA* (**A**), *CXCL12* (**B**), *TGF*β*1* (**C**), *RPGE2* (**D**), *IDO* (**E**), *IL10* (**F**), *IL1*β (**G**), *IL6* (**H**) and *IL32* (**I**) in bone marrow MSC obtained from HC, MDS, AML-MRC and *de novo* AML patients. The “y” axis represents the relative mRNA expression. Horizontal lines indicate medians. The number of samples in each group and *p* values are indicated in the graph. Mann Whitney test.

We next characterized the mRNA expression of cytokines and other molecules in patient-derived MSC and compared these to the HC samples. No significant difference was detected in the MDS-derived MSC group (Fig. 2). AML-MRC-derived MSC showed a significant increase in *IL6* expression (*p* = 0.02). However, d*e novo* AML MSC presented a significant increase in expression levels of *vascular endothelial growth factor A (VEGFA*), *stromal cell-derived factor 1 (CXCL12*), *receptor of prostaglandin E2 (RPGE2*), *indoleamine 2,3-dioxygenase (IDO*), *IL6 and IL32* (all *p *< 0.05), followed by a decrease in *IL10* expression (*p *= 0.009) when compared to the HC group. We also observed a significantly increased expression of *VEGFA, CXCL12, RPGE2* and *IL32* in *de novo* AML, when compared with AML-MRC-derived MSC (*p *< 0.05). There were no differences in the expressions of *TGFβ1* and *IL1β*.

We also analyzed the expression of the four best characterized isoforms of IL32 in our cohort. *De novo* AML MSC presented a significant increase in the expression levels of *IL32γ(p* = 0.01), the IL-32 isoform with the highest biological activity^30^. There were no differences in the expressions of the *IL32α, β* and *δ* transcripts (Supplemental Figure S1).

### Silencing of IL-32 by miRNA and HS5 cell proliferation

**Figure 3:**
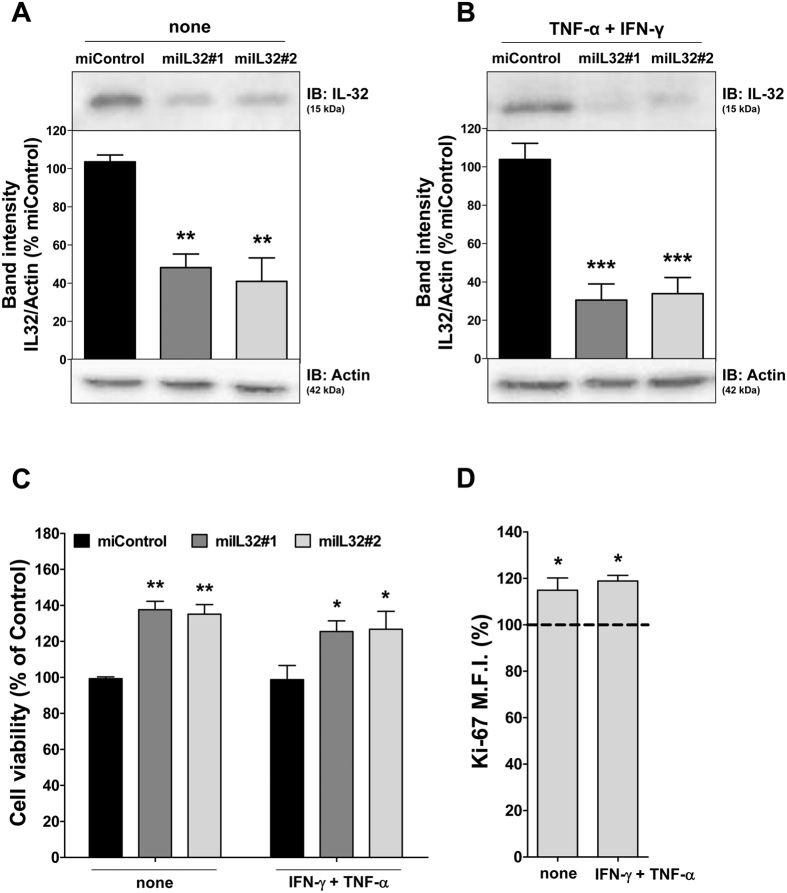
Lentivirus-mediated miRNA efficiently silences IL-32 in HS5 cells and results in increasing of cell proliferation. Western blotting analyses of IL-32 expression for HS5 cells in the presence or not of TNF-α and IFN-γ (10 ng/mL) as indicated. The levels of IL-32 relative to actin were quantified. In the figures are reported the cropped gels/blots. All gels were run in the same experimental conditions (see material and methods for details). (Full-length blots are reported in Supplementary Figure S2). Results are shown relative to miControl cells, as mean ± SEM of three independent experiments. ANOVA, Bonferroni’s post-tests (**p < 0.01; ***p < 0.001). (**C**) Cell viability was determined by MTT assay after 48 hours of incubation of miIL32 (#1 and #2) and normalized by the corresponding miControl cells. Results are shown as mean ± SEM of four independent experiments; The MTT assay was performed in the presence or not of IFN-γ and TNF-α (10 ng/mL) as indicated. ANOVA, Bonferroni’s post-tests (*p < 0.05, **p < 0.01); (**D**) Ki-67 mean of fluorescence intensity (M.F.I.) was determined by flow cytrometry after incubation of miIL32 for 48 h and normalized by the corresponding miControl cells. The dotted line represents the mean of miControl cells. Results are shown as mean ± SEM of four independent experiments; **p < 0.01, Student *t* test.

Several studies have demonstrated that IL-32 plays a role in the inflammatory microenvironment and that there is a network between IL-32 and other cytokines such as IL-1β, IL-6, IL-10 and VEGF^17^,^19^,^21^,^22^,^31^,^32^,^33^. To investigate the relation between IL-32 and MSC, we used HS5, a cell line with a stromal phenotype and with the ability to secret several cytokines, including IL-6 and IL-1, and to support hematopoiesis^34^. Since inflammation triggers MSC activity^35^, we also performed our experiments upon stimulation with IFN-γ and TNF-α. HS5 cells were stably transduced with two lentiviral constructs encoding miRNA targeting *IL32* (miIL32#1 and miIL32#2) or with miControl. After polyclonal cell selection with blasticidin, the efficiency of IL-32 silencing was analyzed by Western blotting. A significant reduction in IL-32 protein levels was observed in both constructions of miIL32 HS5, compared with miControl cells under regular and inflammatory conditions (Fig. 3A and B).

IL-32 is reported to have hematopoietic growth factor properties^36^, while IL-32 silencing results in a reduction in endothelial cell proliferation^37^. To determine whether IL-32 silencing affects stromal cell proliferation and/or viability, MTT assays were performed. Unexpectedly, viability of miIL32 HS5 cells was significantly increased (by ~40%) when compared with miControl cells, with or without pro-inflammatory stimulation (*p *< 0.05; Fig. 3C). Ki-67 analysis revealed that IL-32 silencing significantly increased cell proliferation (Fig. 3D).

### Chemotactic activity of IL-32 on PBMCs

**Figure 4:**
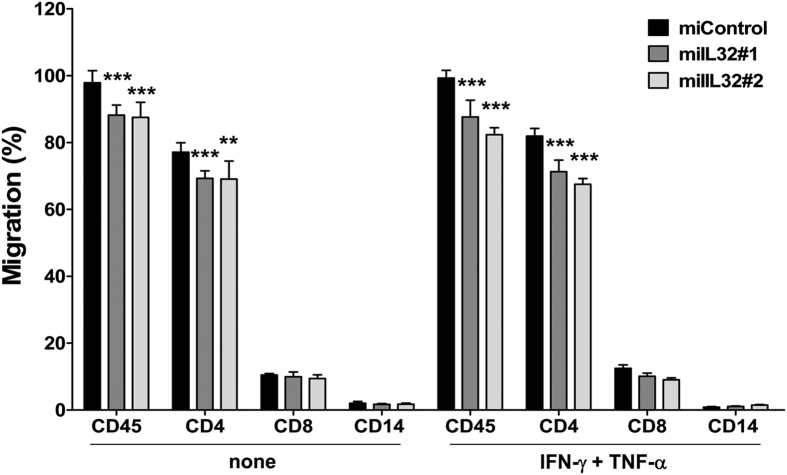
miIL32 HS5 cells have a decreased chemotactic activity on CD4^+^ cells in an inflammatory environment. miIL32 and miControl HS5 cells were seeded in the lower chamber of transwell plates with or without the addition of IFN-γ and TNF-α for 24 h before adding PBMCs, which were allowed to migrate for 5 h. Data represent mean ± SEM (4 independent experiments). ANOVA, Bonferroni’s post-tests (**p < 0.01; ***p < 0.001).

Cell migration is essential for the induction of an effective immune response. To test whether IL-32 has any effect on PBMC recruitment, transwell chemotaxis of PBMC was performed in coculture with miControl or miIL32 HS5 cells. The number and type of PBMC that migrated through the membrane were analyzed. There was a significant decrease in CD45^+^ cells migration, which mainly reflects the significant decrease in CD4^+^ migration, with or without proinflammatory stimulation (*p *< 0.05; Fig. 4).

Similar effects on cell function were observed after IL-32 silencing with both the miIL32 sequences, implying that these effects are the result of RNAi-mediated silencing of the IL-32 gene rather than off-target effects. Since miIL32#2 showed a slightly better efficiency, we chose to carry out the subsequent experiments with this sequence.

### Chemoprotection to AraC conferred by miIL32 HS5 cells

**Figure 5:**
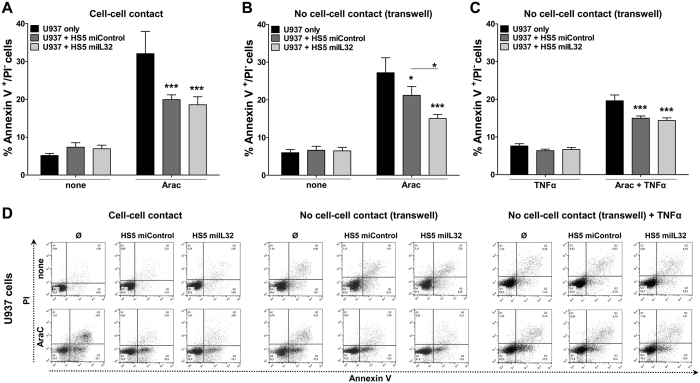
More effective chemoprotection following coculture of U937 cells with miIL32 HS5 cells using transwell chambers. U937 cells were cultured in the presence or absence of miControl or miIL32 HS5 cells in two distinct conditions: (**A**) direct contact or (**B** and **C**) noncontact (using a tranwell system). Cells were allowed to grow for 96 h and AraC was added (1 μM) for the last 18 h of experiment. (**C**) TNF-α was added (10 ng/mL) at the same time as AraC. Apoptosis was assessed by flow cytometry analysis of Annexin-V-APC/PI–stained cells. Data represent means ± SEM (6 independent experiments). ANOVA, Bonferroni’s post-tests (*p < 0.05; **p < 0.01; ***p < 0.001). (**D**) Dot plots are representative of one experiment.

We firstly evaluated whether IL-32 silencing could modify the chemoprotection to AraC-induced apoptosis, the most effective drug for the treatment of AML^38^, conferred by HS5 to U937 cells^39^. As observed in Fig. 5A, IL-32 silencing does not modify the resistance of U937 cells to AraC when they are in contact with HS5 (*p *< 0.001).

Next, we tested whether the supernatant of miIL32 HS5 cells could change the chemosensitivity of U937 cells to AraC, using a transwell system. In these conditions, we observed that HS5 cells still protect U937 from AraC cytotoxicity. However, noncontact with miIL32 HS5 was significantly more effective than the miControl in inhibiting AraC-induced apoptosis of U937 cells (*p *< 0.05; Fig. 5B and D). Interestingly, this effect was reverted by the addition of TNF-α (Fig. 5C and D).

### Modulation of cytokines, chemokines, MAPK and NF-κB signaling by IL-32

**Figure 6:**
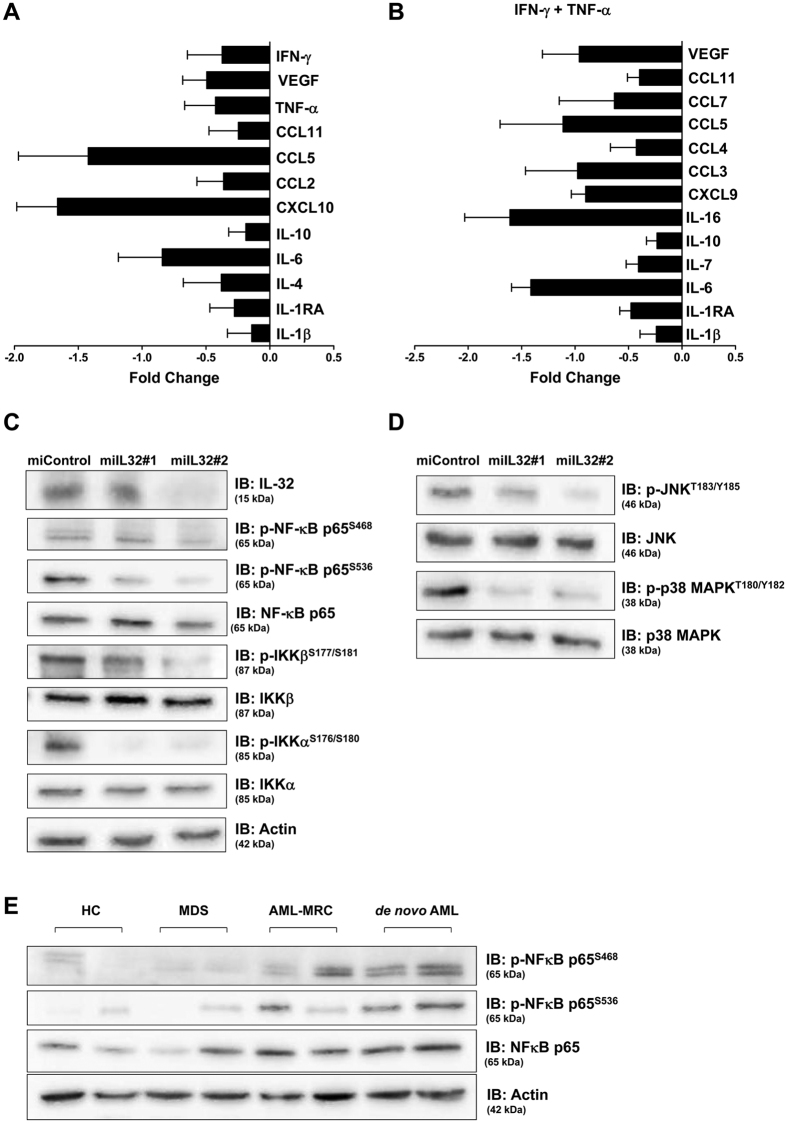
IL-32 silencing down-modulates cytokines, MAPK and NF- κB signaling components in HS5 cells. (**A**,**B**) The fold-change in cytokine concentration in miIL32 HS5 cell supernatant compared to miControl cell values is plotted, in the presence or not of IFN-γ and TNF-α (10 ng/mL) as indicated. Results are shown as mean ± SEM of 3 independent experiments. (**C**) HS5 cells transduced either with miIL32#1 or miIL32#2 and miControl. Western blot for IL-32, p-NF-κB, p-IKKβ, and p-IKKα; (**D**) p-JNK and p-p38 MAPK. (**E**) Activation status of NF-κB signaling in AML-MSC patients; western blot for p-NF-κB in total protein of MSC cells. Gels were run under the same experimental conditions while images of western blots displayed in cropped format. Membranes were reprobed with antibodies against actin or total protein. Full-length blots/gels are presented in Supplementary Figure S3.

We also measured, in serum-free supernatants from cell culture of miControl and miIL32, the levels of cytokines and chemokines, in order to assess whether IL-32 regulates their expression in the stromal cell. The major cytokines downmodulated by IL-32 are demonstrated in Fig. 6A and B.

The ability of IL-32 to activate the p38 mitogen-activated protein kinase (MAPK) and nuclear factor-kappa B (NF-κB) pathways has been previously reported^17^,^40^,^41^. Herein, we sought to examine the effect of IL-32 on MAPK and NF-κB signaling in our stromal cell line model. We observed that IL-32 depletion (Fig. 6C) resulted in a decrease in phosphorylation of NF-κB, IKKα, IKKβ, c-Jun N-terminal kinase (JNK) and p38 MAPK (Fig. 6C and D). Furthermore, we analyzed the phosphorylation levels of NF-κB in samples from patient-derived MSC and HC-derived MSC. In this small set of patient-derived cells, NF-κB phosphorylation levels were higher in the AML-derived MSC, especially in *de novo* AML samples (Fig. 6E). This finding is worthy of further research.

## Discussion

In this report, we characterized and compared MSC derived from patients with MDS, AML-MRC and *de novo* AML with these from healthy controls. Although MSC from patients exhibited a typical antigen expression profile, as already described by others^42^, we observed a reduced immunosuppressive ability of AML-MSC when compared to the control. There are few studies about the immunosuppressive ability of AML-derived MSC^43^, although studies about MDS-MSC immunosuppressive function have been controversial^43^,^44^,^45^,^46^. We found that MDS-MSC were capable of satisfactorily inhibiting T cell proliferation *in vitro* induced by PHA, even when we classified our cohort as low or high-risk MDS, according to the WHO 2008 classification^2^. Supporting this finding, we observed no significant difference in any cytokine or molecule studied in the MDS-MSC. In agreement with Klaus *et al*.^44^, our data suggest that MDS-derived MSC are not the main factor responsible for the aberrant T cell response, sometimes observed in MDS patients.

We also demonstrated a distinct cytokine profile between AML-MRC and *de novo* AML-derived MSC, suggesting that the difference between these two AML subtypes also extends into the leukemic stem cell niche. AML-MRC is characterized by the persistence of the malignancy, alterations in the hematopoietic niche caused by prior MDS treatment, a higher frequency of molecular mutations and cytogenetic abnormalities^47^. We observed that, despite the impaired immunosuppressive ability of the AML-MRC-derived MSC, their cytokine profile is very similar to that of the control group. Conversely, *de novo* AML-derived MSC presented a significant increase in their expressions of *VEGF* and *IL6*, both of which are secreted by the leukemic blast in order to promote their survival and proliferation^48^,^49^,^50^,^51^. Recently, Kim *et al*.^52^ demonstrated that leukemia stem cells from *de novo* AML patients can induce extensive alterations in the mesenchymal niche, resulting in an altered expression of crosstalk molecules, including CXCL12. Together with the lower *IL10* expression^53^,^54^, and the higher expression of *IDO*^55^,^56^,^57^, *CXCL12*^58^,^59^,^60^ and *RPGE2*^61^,^62^,^63^ observed in our *de novo* AML cohort, we suggest that at least at the beginning of this disease, prior to any treatment, MSC provides a permissive niche for leukemogenesis rather than normal hematopoiesis. Marcondes *et al*.^22^ showed an increase in *IL32* expression in the bone marrow stromal cells from 13 patients with MDS. Nevertheless, in our cohort of 22 MDS patients we could not see any statistical difference between the MDS and HC groups. These differences may due to either a lack of standardized culture for bone marrow MSC or the heterogeneity of MDS patients. Furthermore, in *de novo* AML-derived MSC, we observed a significantly higher expression level of *IL32*, which reflects the expression of the most active isoform, *IL32γ*. It has been shown that the over expression of IL-32γ in an animal model resulted in the inhibition of the cell proliferation of melanoma and colon tumors^64^. Our results suggest that the higher expression of *IL32γ* in *de novo* AML-derived MSC could contribute to the slower-growing cells generated from these patients samples, as observed in our study. Accordingly, our *in vitro* results show that IL-32 inhibition resulted in a significantly increased stromal cell proliferation, with our without proinflammatory stimulation. IL-32 may have pleiotropic effects on cell proliferation, as indicated by many other cellular functions^65^,^66^,^67^,^68^,^69^,^70^.

Son *et al*. demonstrated that endogenously-secreted IL-32γ may regulate a variety of immune responses via CCL5 expression from dendritic cells, including chemotaxis of activated T cells^71^. We observed that silencing of IL-32 resulted in lower concentrations of CCL5 in the supernatant of HS5 cells, which may explain the significantly decreased migration of CD4^+^ cells when cocultured with HS5 miIL32 cells. Additionally, many other important inflammatory molecules, including VEGF, TNF-α, IFN-γ, and IL-6 were reduced in the supernatant of HS5 miIL32 cells.

Our study on AraC-mediated cytotoxicity has shown that coculture of HS5 miControl or miIL32 with U937 cells *in vitro* resulted in the same levels of enhanced protection compared to U937 without HS5 cells. However, the coculture of HS5 miIL32 with U937 cells using transwell assays resulted in a significant protection from AraC-induced apoptosis when compared to the coculture with HS5 miControl. It has been described that HS5 cells can secrete a soluble factor(s) that protect U937 from AraC induced apoptosis^72^. Herein, we showed that IL-32 has a role in stromal cell crosstalk with leukemia cells, and that TNF-α must be part of this network, at least *in vitro*, since the protection from AraC-induced apoptosis conferred by HS5 miIL32 was reversed upon the addition of TNF-α. In a recent studies, it was shown that IL-32α suppressed colon cancer development by promoting the death signaling of TNFR1^73^, and that IL-32γ can induce TFN-α production in differentiated THP1 cell line^74^.

Proliferation, leukocyte chemotaxis and regulation of the apoptotic threshold depend on appropriate signals through a favorable cytokine milieu for their homing to the bone marrow^75^,^76^,^77^,^78^. Our results suggest that IL-32 participates in the regulation of the bone marrow cytokine milieu, at least in part, through MAPK and NF-κB signaling.

In conclusion, we have shown a distinct function and cytokine profiles in AML-MRC and *de novo* AML-derived MSC, including the expression of IL-32. We also demonstrate that IL-32 takes part in the chemoresistance induced by MSC.

## Materials and Methods

### Primary samples

For isolation and expansion of MSC, bone marrow cells were obtained from 8 healthy donors (healthy control; HC) and 22 MDS and 19 AML patients. For the T cell proliferation and chemotaxis assay, peripheral blood mononuclear cells (PBMC) from HC (n = 26) were obtained by Ficoll-Hypaque gradient separation. All HC and patients provided their informed written consent, and all experiments were performed in accordance with the ethical and care guidelines and were approved by the local ethics committee of the University of Campinas and was adherent to the Declaration of Helsinki. Patients were untreated at the time of sample collection. Patients’ characteristics are described in Supplementary Table 1. MDS patients were classified according to the WHO 2008 classification^2^. Acute promyelocytic leukaemia [APL or AML with t(15;17)(q22;q12)] was excluded from the *de novo* AML group.

### Cell lines and chemical reagents

The human bone marrow stromal cell line, HS5, and the human acute myeloid leukemia cell line, U937^79^, were obtained from the American Type Culture Collection (Manassas, VA, USA). 293FT and HT1080 cells were acquired in 2012 from Invitrogen (Carlsbad, CA, USA). Cells were cultured in appropriated medium, according to the manufacturer’s instruction, containing 10% fetal bovine serum (FBS) and glutamine with penicillin/streptomycin and amphotericin B, and maintained at 37 °C, 5% CO_2_. Recombinant human TNF-α and IFN-γ were purchased from PeproTech (Rocky Hill, NJ, USA). Cytarabine (AraC) was obtained from Intas Pharmaceuticals (Ahmedabad, India) and prepared as a 10 mM stock solution.

### Isolation and expansion of MSC from bone marrow

Mononuclear cells were isolated from bone marrow by density gradient centrifugation and seeded at a density of 10^6 ^cells/cm^2^. After 3–4 days of adhesion, non-adherent cells were removed. Cells were cultured at 37 °C, 5% CO_2_ in DMEM medium containing 1% penicillin/streptomycin, 1% L-glutamine and 10% fetal bovine serum, as described previously^80^. After achieving 80% confluence, the cells were removed with trypsin and plated again at a concentration of 4 × 10^3^ cells/cm^2^, for further passages. We performed all analyses at the fourth passage.

### Quantitative PCR (q-PCR)

Total RNA was purified using the TRIzol Reagent (Invitrogen). The reverse transcription reaction was performed using RevertAid First Strand cDNA synthesis kit (MBI Fermentas, Amherst, NY, USA). Expression of mRNAs was detected by qPCR with the Maxima SYBR Green qPCR master mix (MBI Fermentas) using the ABI 7500 Sequence Detection System (PE Applied Biosystems, Foster City, CA, USA). *HPRT* was used as an endogenous control (sequences of all genes at Supplementary Table 3). The relative quantification value was calculated using the equation, 2^−ΔΔCT^ 81.

### Flow cytometry

Immunophenotyping of MSC was evaluated by flow cytometry (FACSCalibur, Becton-Dickinson, San Jose, CA, USA). Fluorochrome conjugated monoclonal antibodies, anti-CD45-FITC or -PercP, CD34-APC, CD73-PE, CD31-FITC^27^, HLADR-FITC, CD90-PeCy5, and CD105PE. The U937 leukemia cell line was also stained for the detection of apoptotic cells (Annexin-V-APC positive/propidium iodide (PI) negative populations) after coculture with HS5 cells. Analyses were performed using the FACSDiva software (version 4.0.1, Becton-Dickinson) or FlowJo software (Treestar, Inc., San Carlos, CA, USA).

### Fibroblastic colony-forming unit (CFU-F) assay

CFU-F assays were performed by plating 1 × 10^3^ cells/well in 6-well plates. The medium was changed on day 7. After 15 days of culture, adherent cells were washed twice with phosphate-buffered saline, and stained with 1% crystal-violet in methanol. CFU-F colonies were macroscopically enumerated and clusters of more than 50 cells were considered as colonies.

### T cell proliferation assay

CD3^+^ cells were purified from isolated PBMC, by magnetic separation of bead-bound cells (Miltenyi Biotec GmbH, Bergisch Gladbach, Germany). Purity of CD3^+^ cells was 90–95%, as assessed by flow cytometry. CFSE-labeled CD3^+^ cells were resuspended and added to wells (10^5^ cells/well) containing MSC at MSC/T cells ratios of 1:2, 1:5, 1:10, 1:50 and 1:100 in the presence of PHA (2,5 μg/mL). Four days later the CFSE fluorescence intensity was analyzed by flow cytometry (Becton-Dickinson).

### Preparation of lentiviral vectors

Lentiviral vectors, expressing microRNA (miRNA) and targeting human IL-32 or LacZ were prepared using the BLOCK-iT Pol II miR RNAi Expression Vector with EmGFP System (Invitrogen), following the manufacturer’s instructions. The target sequence used to silence LacZ was 5′-AAATCGCTGATTTGTGTAGTC-3′. Two distinct sequences to silence IL-32 were used; sequence 1 (5′-AGAGGGCTACCTGGAGACAGT-3′) and sequence 2 (5′-GAGACAGTGGCGGCTTATTAT-3′). Viral concentrations were determined by *in vitro* transduction in HT1080 cells and using blasticidin selection.

### Transduction of lentivirus

HS5 cells were transduced with lentivirus-mediated miRNA targeting LacZ (named miControl) or lentivirus-mediated miRNA targeting IL-32; miIL32#1 for sequence 1 and miIL32#2 for sequence 2. Briefly, HS5 cell lines were seeded onto six-well plates at 5 × 10^4 ^cells/well, grown overnight, and transduced with lentiviral vectors at a multiplicity of infection equal to 1 in a minimal volume of medium containing 6 mg/mL of polybrene (Sigma-Aldrich, St. Louis, MO, USA). The transduced cells were selected for 15 days using blasticidin (10 μg/mL) before functional analyses.

### Western blotting

Western blot analysis was performed as described previously^82^. Anti-IL-32 (sc-134446) and actin (sc-1616) antibodies were obtained from Santa Cruz Biotechnology. Anti-phospho-SAPK/JNK (Thr183/Tyr185; #9251), SAPK/JNK (9252), phospho-p38 MAPK (Thr180/Tyr182; 9211), p38 MAPK (8690), phospho-NF-κB p65 (Ser468; 3039), NF-κB p65 (4764), phospho-IKKα/β (Ser176/180; 2697), IKKα (2682) and IKKβ (8943) were obtained from Cell Signaling. Quantification of band intensity was performed by UN-SCAN-IT (Silk Scientific, Orem, UT, USA).

### Cell viability of HS5 cells

Cell viability was measured by methylthiazoletetrazolium (MTT) assay. After 16 h of serum starvation, cells were stimulated to reenter the cell cycle and to proliferate using DMEM supplemented with 10% FBS. Serum starvation was not toxic to the cells (evaluated by Trypan blue; data not shown). A total of 9 × 10^3^ cells per well were plated in 96-well plates in DMEM 10% FBS in the absence or presence of proinflammatory cytokines (10 ng/mL TNF-α and 10 ng/mL IFN-γ) for 48 h. In brief, 10 μL of a 5 mg/mL solution of MTT was added to the wells and incubated at 37 °C for 4 h. The reaction was stopped using 100 μL of 0.1 N HCl in anhydrous isopropanol. Cell viability was evaluated by measuring the absorbance at 570 nm, using an automated plate reader. All conditions were tested in six replicates.

### Cell proliferation by Ki-67 staining

After 16 h of serum starvation, cells were stimulated to reenter the cell cycle and to proliferate using DMEM supplemented with 10% FBS, exposed or not to the proinflammatory environment (10 ng/mL TNF-α and 10 ng/mL IFN-γ). Ki-67 staining was performed following the manufacturer’s instructions (Ki-67 APC clone B56; Becton-Dickinson) and the mean fluorescence intensity (M.F.I.) was obtained by flow cytometry (FACSCalibur, Becton Dickinson). An IgG isotype was used as a negative control for each condition. Ten thousand events were acquired for each sample.

### Chemotaxis assay

We performed a coculture of miControl or miIL32 HS5 cells and PBMC using a transwell culture system (8 μM-pore size membrane, Corning, NY, USA). HS5 cells were seeded in the lower chamber in DMEM/0.3% BSA exposed or not to an inflammatory environment (10 ng/mL TNF-α and 10 ng/mL IFN-γ). After 24 h, PBMC were seeded in the upper chamber (10^5 ^cells/well). After 5 h, migrated cells were analyzed; cells from the lower chamber were trypsinized, stained with anti-CD45, anti-CD14, anti-CD4, and anti-CD8 and analyzed by flow cytometry. Results were calculated as the percentage of the 100% migration value (input).

### Chemosensitivity assay to AraC

U937 cells were cocultured with miControl or miIL32 HS5 cells in two distinct conditions: direct contact or noncontact (using a 0.4 μm porous transwell insert that allows passage of soluble growth factors). Cells were allowed to grow for 96 h and AraC (1 μM) with or without TNF-α (10 ng/mL) was added in the last 18 h of experiment. Cells were collected and submitted to Annexin-V-APC/PI double staining for flow cytometry. U937 cells were distinguished from miControl or miIL32 HS5 cells by a gate in GFP-positive cells. The analysis was performed using FACSDiva software (version 4.0.1, Becton-Dickinson).

### Bio-Plex human cytokine quantification assay

Culture supernatants from miControl and miIL32 HS5 cells, exposed or not to the proinflammatory environment (10 ng/mL TNF-α and 10 ng/mL IFN-γ), were assayed for several cytokines, chemokines and growth factors using a Bio-Plex human cytokine 27-plex and 21-plex panel assay (Bio-Rad, Hercules, CA, USA). Samples were tested according to the manufacturer’s instruction. Data were collected and analyzed using a Bio-Rad BioPlex 200 instrument equipped with Bio-Plex Manager software version 6.0 (Bio-Rad Laboratory, Hercules, CA, USA).

### Statistical analysis

Statistical analysis was performed using GraphPad Prism 5 (GraphPad Software, San Diego, CA, USA). For comparisons, Mann-Whitney or Student t tests were used for measured factors with 2 levels; ANOVA followed by post-hoc Bonferroni was used for measured factors with 3 or more levels. A *p* < 0.05 was considered as statistically significant.

## Additional Information

**How to cite this article**: Lopes, M. R. *et al*. *De novo* AML exhibits greater microenvironment dysregulation compared to AML with myelodysplasia-related changes. *Sci. Rep.* **7**, 40707; doi: 10.1038/srep40707 (2017).

**Publisher's note:** Springer Nature remains neutral with regard to jurisdictional claims in published maps and institutional affiliations.

## Supplementary Material

Supplementary Information
