# Supplementary material for: De novo AML exhibits greater microenvironment dysregulation compared to AML with myelodysplasia-related changes
Source: Sci Rep. 2017 Jan 13;7:40707. doi: 10.1038/srep40707 (PMC5234038; doi:10.1038/srep40707)
Supplement: Supplementary Information [file srep40707-s1.pdf]

De novo AML exhibits greater microenvironment dysregulation compared to AML with myelodysplasia-related changes

Matheus Rodrigues Lopes[1]; João Kleber Novais Pereira[1]; Paula de Melo Campos[1]; João Agostinho Machado-Neto[1]; Fabiola Traina[1,2]; Sara T. Olalla Saad[1]; Patricia Favaro[1,3, *]

***corresponding author:** favaropb@gmail.com, patricia.favaro@unifesp.br

Supplementary Table 1. Patients' characteristics.

**Patients**	**Number**
**MDS**	22
Male/Female	16/06
Age (yr), median (range)	71 (16-90)
WHO classification	
RCUD/RCMD/RARS	01/11/04
RAEB-1/RAEB-2	02/04
**AML**	19
Male/Female	08/11
Age (yr), median (range)	64 (30-86)
AML with MDS-related changes	07
de novo AML	12

HC, healthy controls; MDS, myelodysplastic syndrome; AML, acute myeloid leukemia; RCMD, refractory cytopenia with multilineage dysplasia; RARS, refractory anemia with ring sideroblasts; RAEB-1/2, refractory anemia with excess blasts-1/2.

Supplementary Table 2. Pattern of markers differentially expressed on MSCs. Percentage of positive cells (mean ±SD).

CD marker	HC	MDS	AML-MRC	*de novo* AML
**CD90**	98.3 (± 2.3)	97.6 (± 4.0)	99.3 (± 0.8)	94.8 (± 4.4)
**CD105**	94.1 (± 5.8)	94.8 (± 5.2)	97.1 (± 4.3)	93.0 (± 7.2)
**CD73**	87.7 (± 11.5)	86.8 (± 16.5)	94.3 (± 6.5)	76.3 (± 26.3)
**HLA DR**	1.3 (± 0.7)	1.4 (± 0.8)	3.0 (± 1.4)	2.8 (± 1.7)
**CD34**	3.9 (± 2.6)	6.2 (± 5.7)	13.0 (± 9.7)	2.8 (± 2.5)
**CD45**	1.2 (± 0.4)	1.6 (± 1.8)	2.0 (± 0.9)	2.8 (± 1.9)
**CD31**	1.8 (± 1.3)	2.1 (± 1.4)	10.3 (± 15.8)	2.1 (± 1.4)

HC. healthy control; MDS. myelodysplastic syndrome; AML. acute myeloid leukemia; AML-MRC. AML with MDS-related changes. Mann Whitney test.

Supplementary Table 3. Sequence and concentration of primers used for qPCR.

Gene	Foward primer	Reverse primer	Concentration
IL32	GAACTTTTGGCCGCCATGT	GGGCCTTCAGCTTCTTCATGT	150 nM
IL32α	GACAGTGGCGGCTTATTATGAG	GCTCCGTAGGACTTGTCACAAA	150 nM
IL32β	CTGTCTCTCTCGGCTGAGTATTTGT	TCTTCATGTCATCAGAGAGGACCTT	300 nM
IL32γ	GTAATGCTCCTCCCTACTTC	GCAAAGGTGGTGTCAGTATC	300 nM
IL32δ	GACGTGGACAGGACGACTTCA	CCTCGGCACCGTAATCCAT	150 nM
VEGFA	AGCCTTGCCGCCTTGCTGCTCTA	GTGCTGGCCTTGGTGAGG	300 nM
CXCL12	GAGCTACAGATGCCCATGC	CTTTAGCTTCGGGTCAATGC	150 nM
IDO	TTGGAGAAAGCCCTTCAAGTG	TGCCTTTCCAGCCAGACAA	300 nM
RPGE2	CCCCCAGTATTGCAGGAG	TAGACGAAGCCCAGGAAAAG	150 nM
IL10	GCTGAGAACCAAGACCCAGA	AAATCGATGACAGCGCCGT	300 nM
TGFβ1	GCGTGCTAATGGTGGAAACC	GCTTCTCGGAGCTCTGATGTG	150 nM
IL1β	CTTTGAAGCTGATGGCCCTAAA	AGTGGTGGTCGGAGATTCGT	300 nM
IL6	GGTACATCCTCGACGGCATCT	GTGCCTCTTTGCTGCTTTCAC	150 nM
HPRT	GAACGTCTTGCTCGAGATGTGA	TCCAGCAGGTCAGCAAAGAAT	150 nM

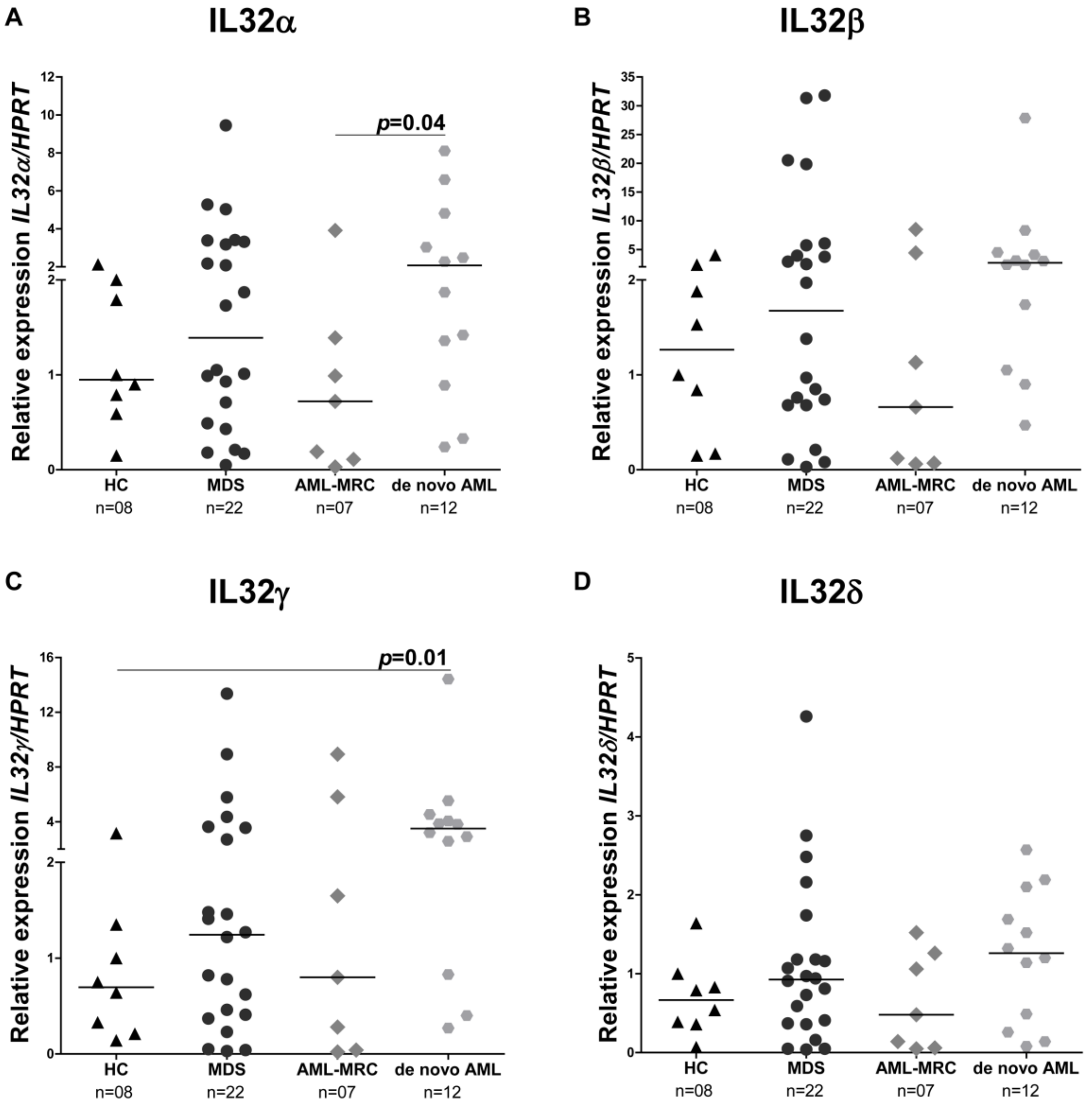

SUPPLEMENTARY FIGURE S1. Expression of isoforms of IL32. qPCR analyses of mRNA expression of *IL32*α (A), *IL32*β (B), *IL32*γ (C), and *IL32*δ (D), in bone marrow MSCs obtained from HC, MDS, AML-MRC and *de novo* AML patients. The "y" axis represents the relative mRNA expression. Horizontal lines indicate medians. The number of samples in each group and *p* values are indicated in the graph. Mann Whitney test.

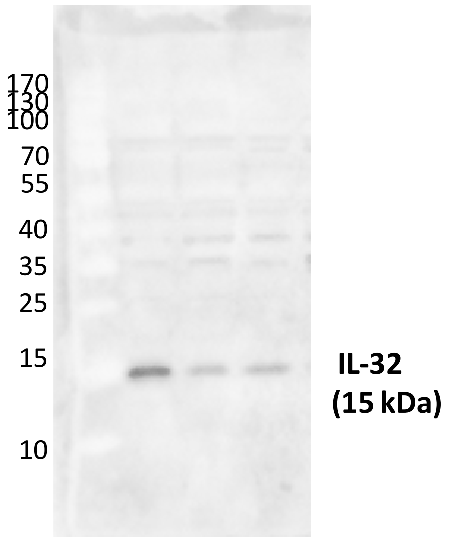
170
130
100
70
55
40
35
25
15
10
IL-32
(15 kDa)

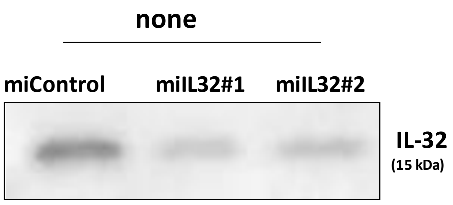
none
miControl
miIL32#1
miIL32#2
IL-32
(15 kDa)

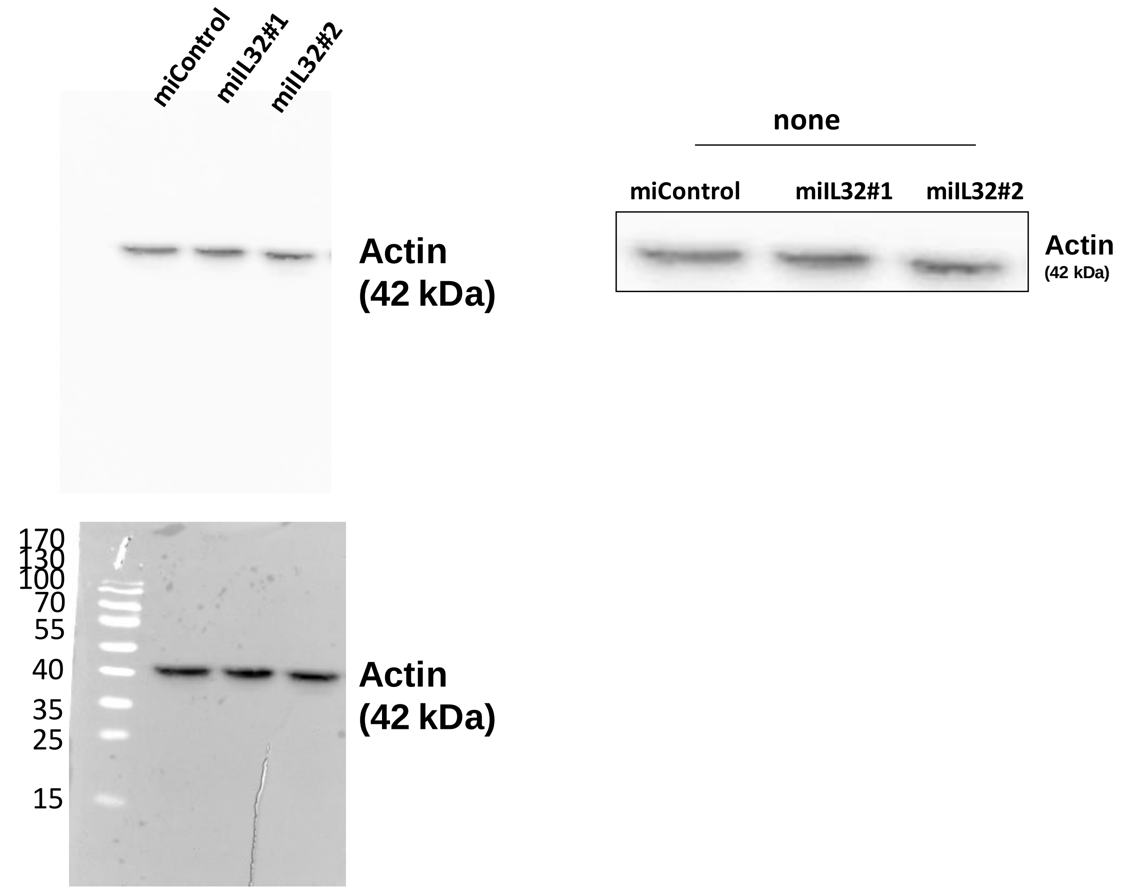

SUPPLEMENTARY FIGURE S2

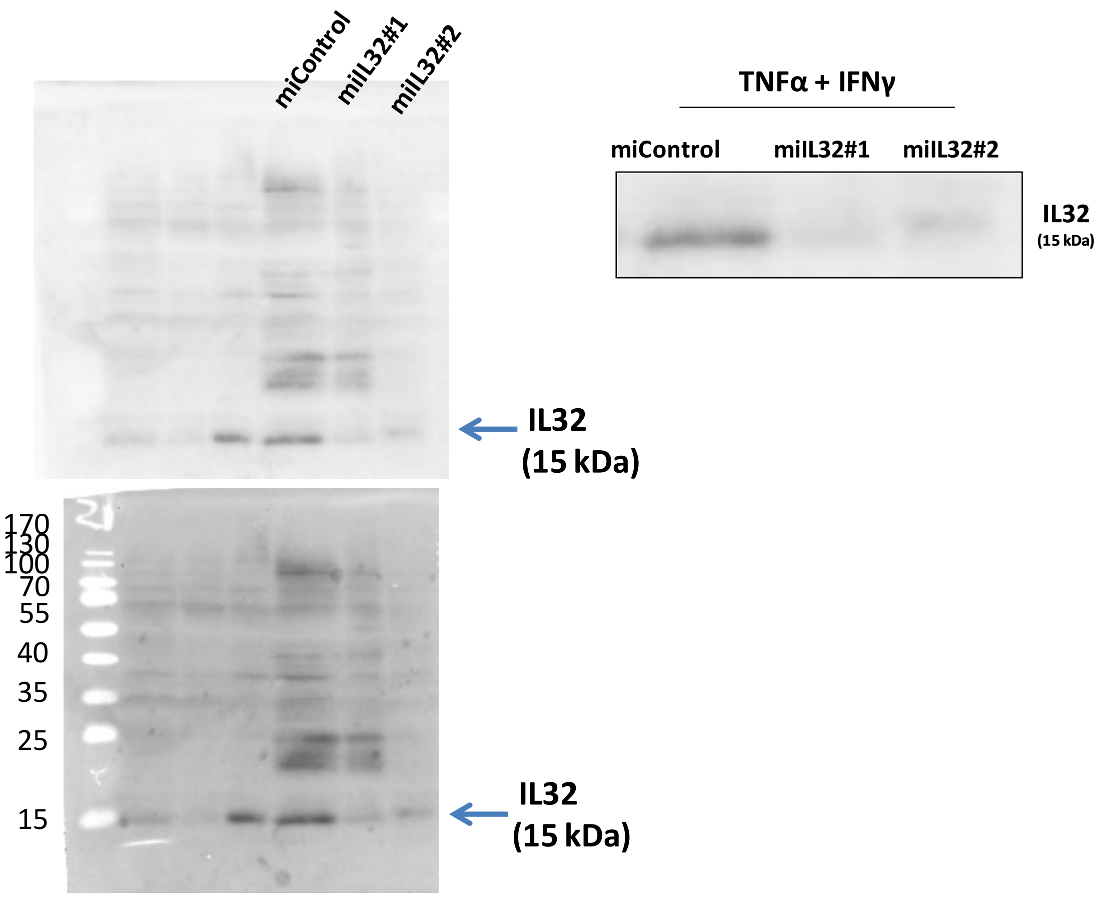

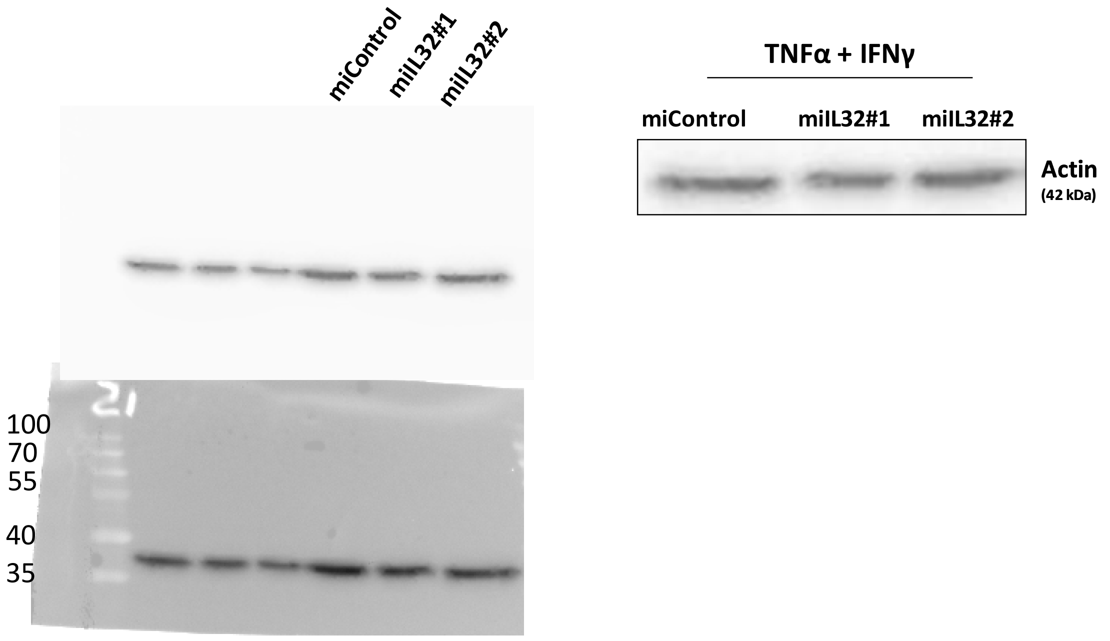

SUPPLEMENTARY FIGURE S2

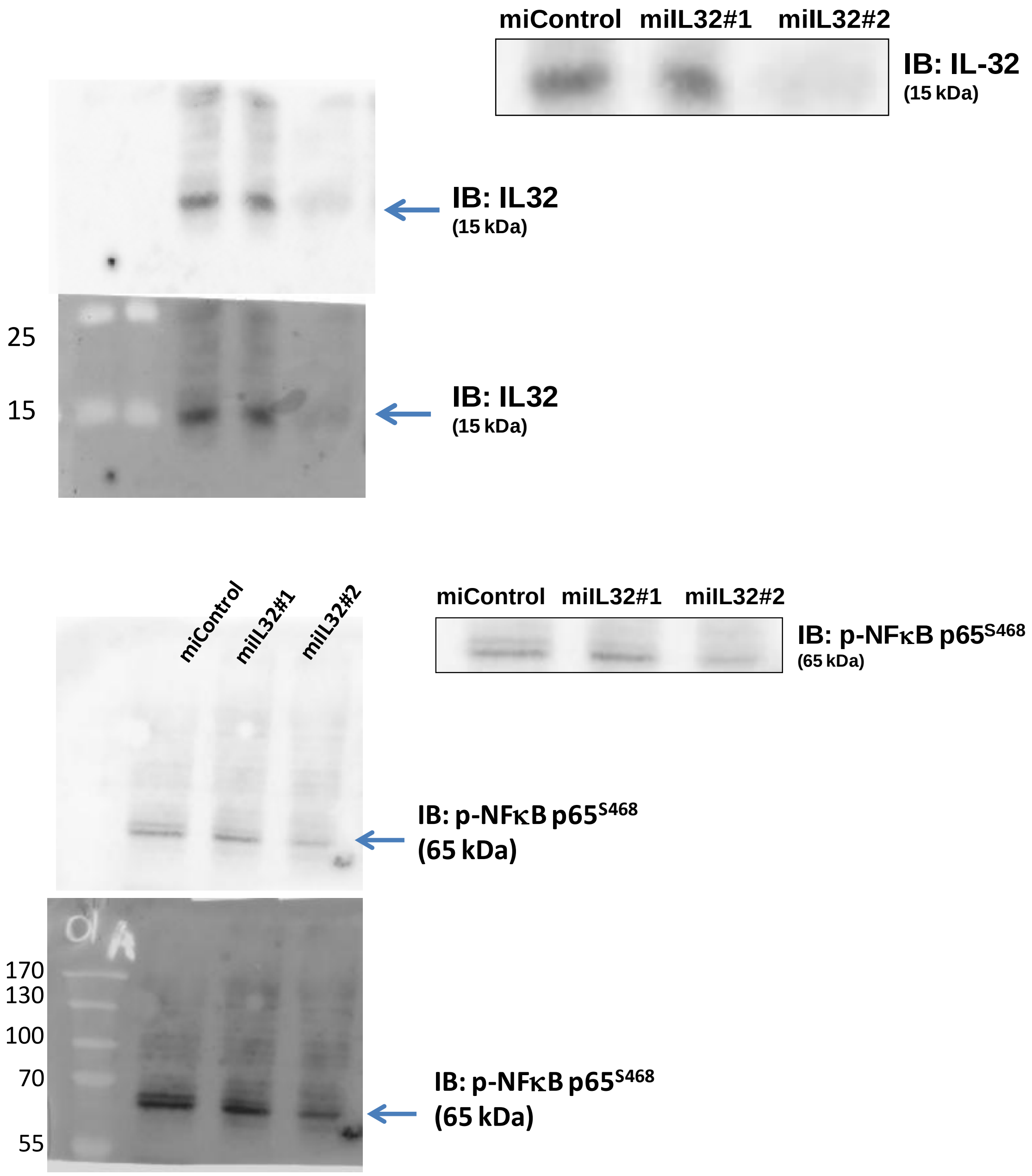

SUPPLEMENTARY FIGURE S3

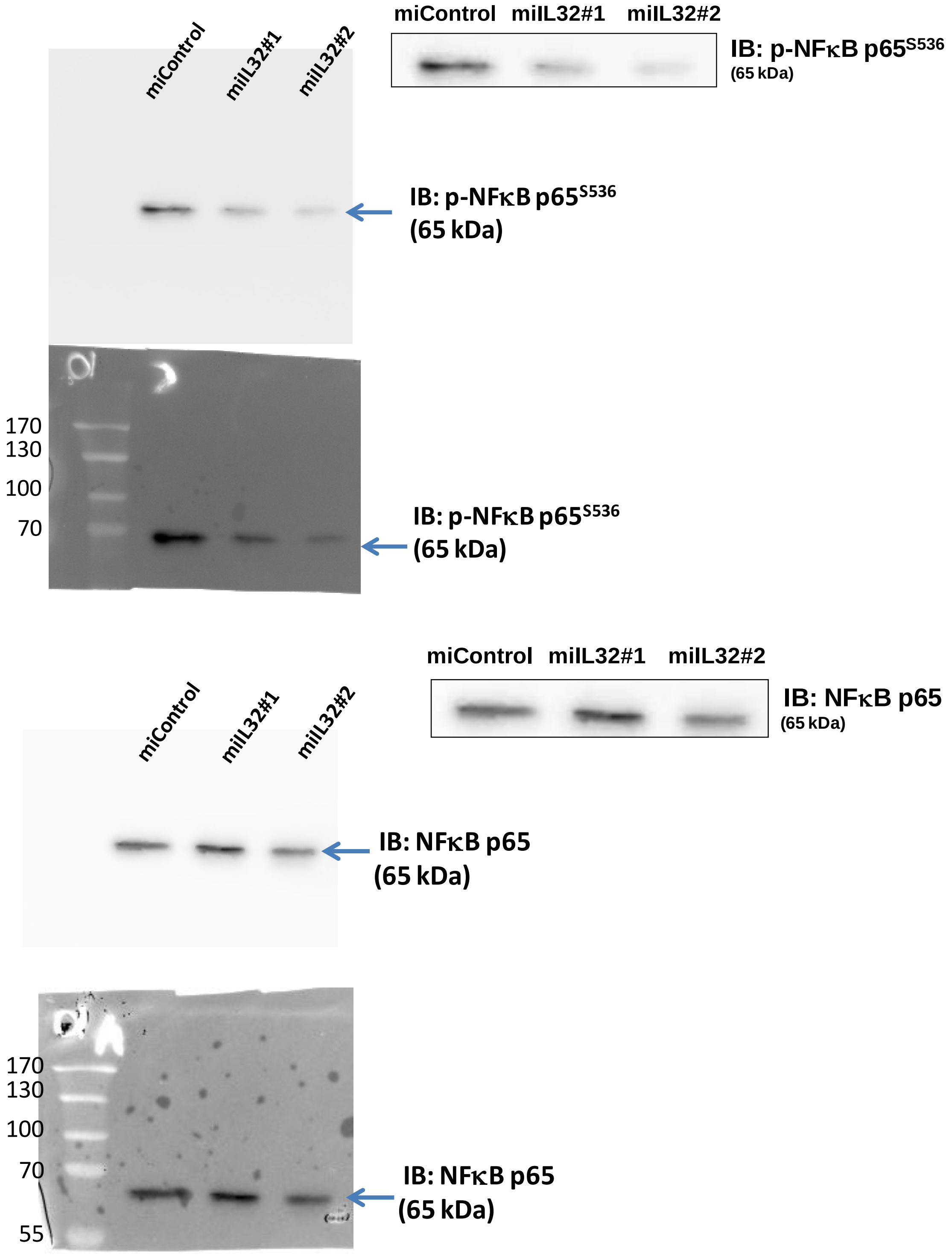

SUPPLEMENTARY FIGURE S3

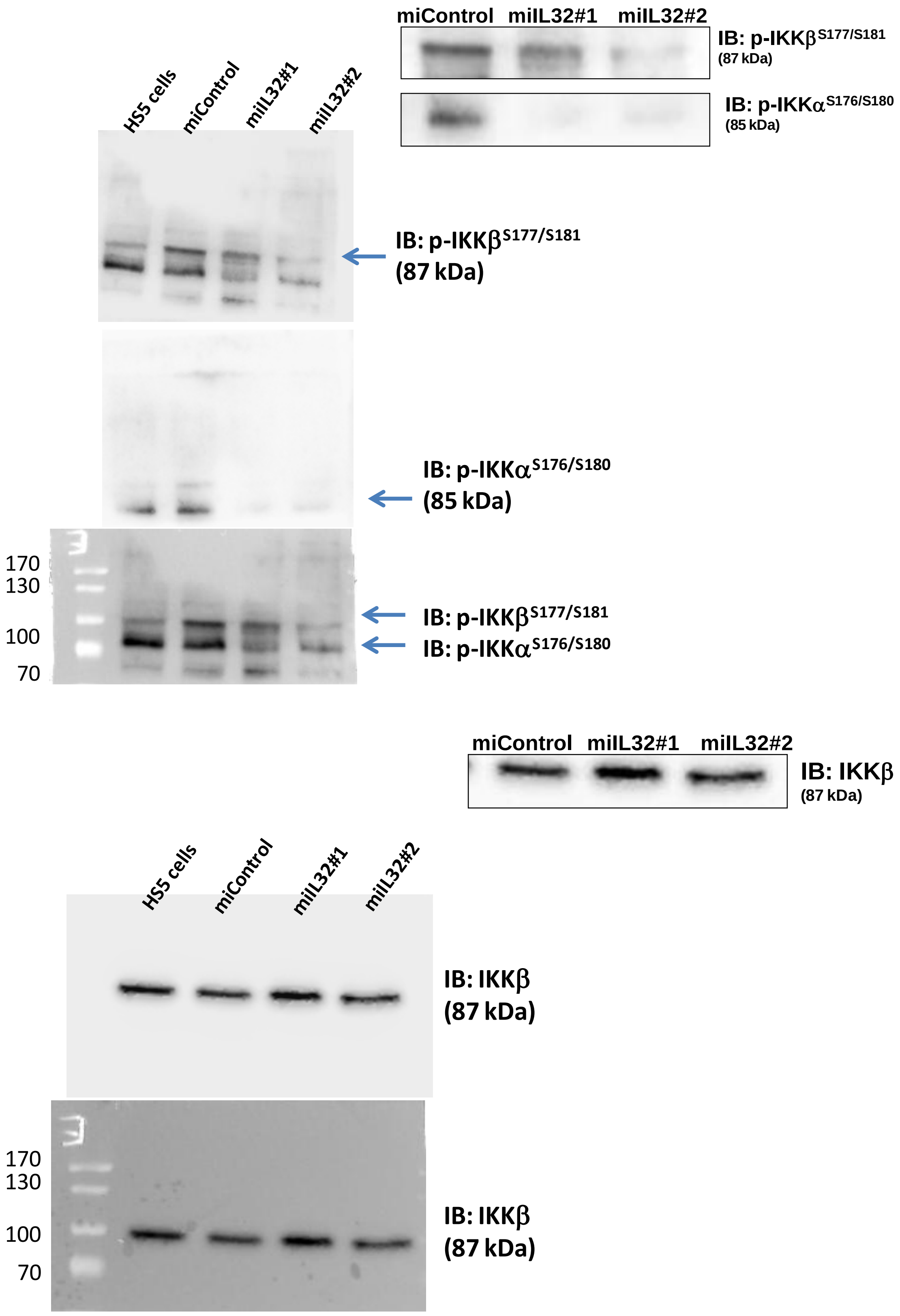

SUPPLEMENTARY FIGURE S3

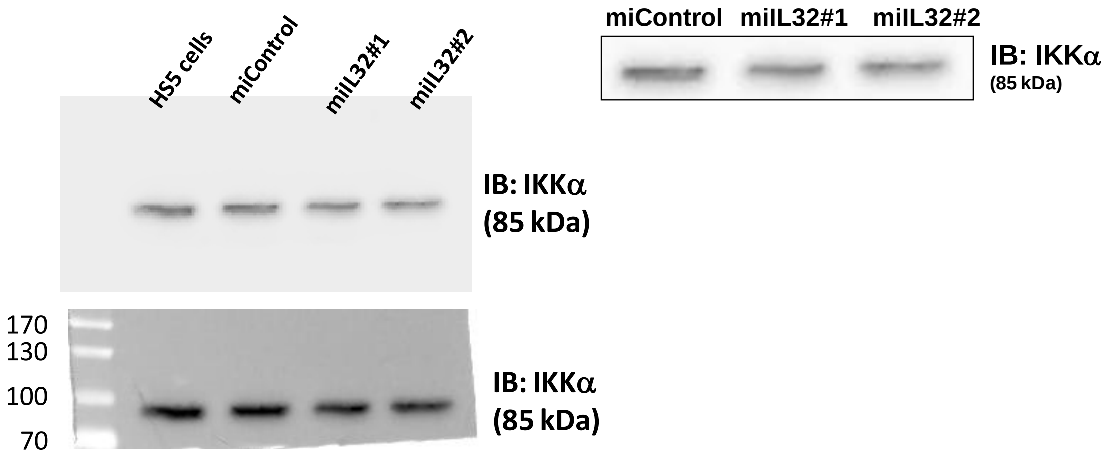

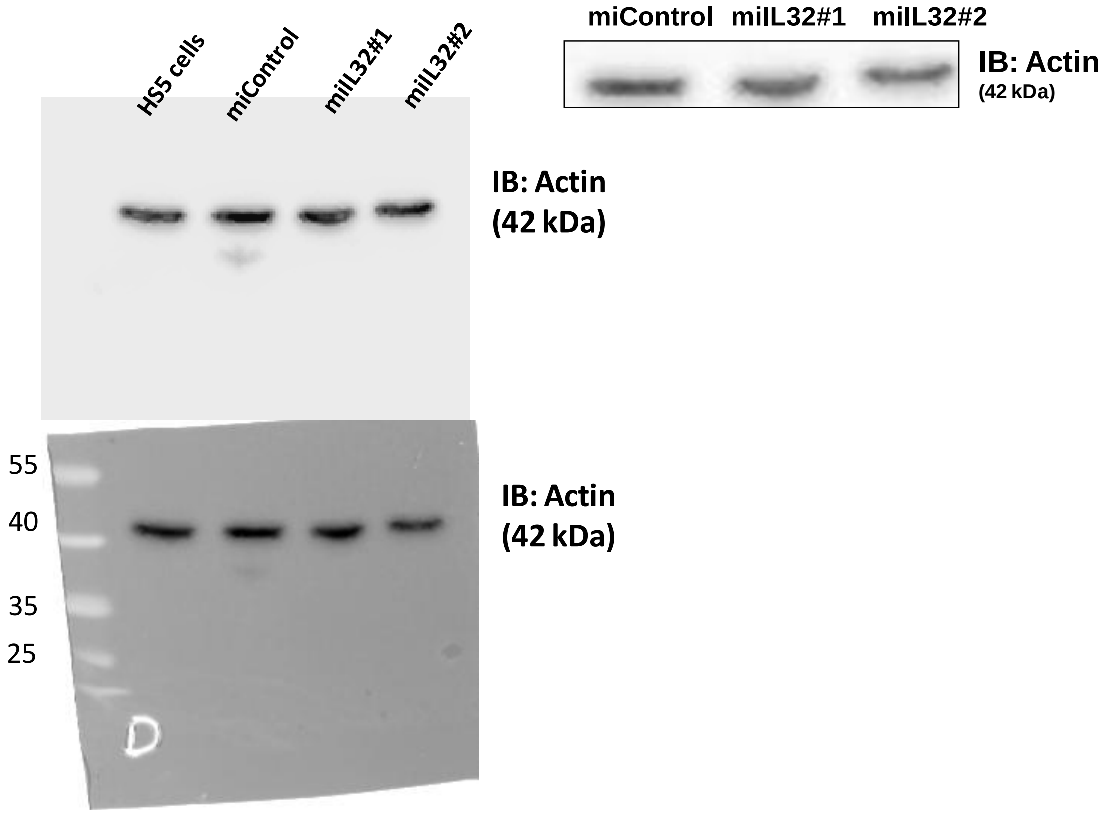

SUPPLEMENTARY FIGURE S3

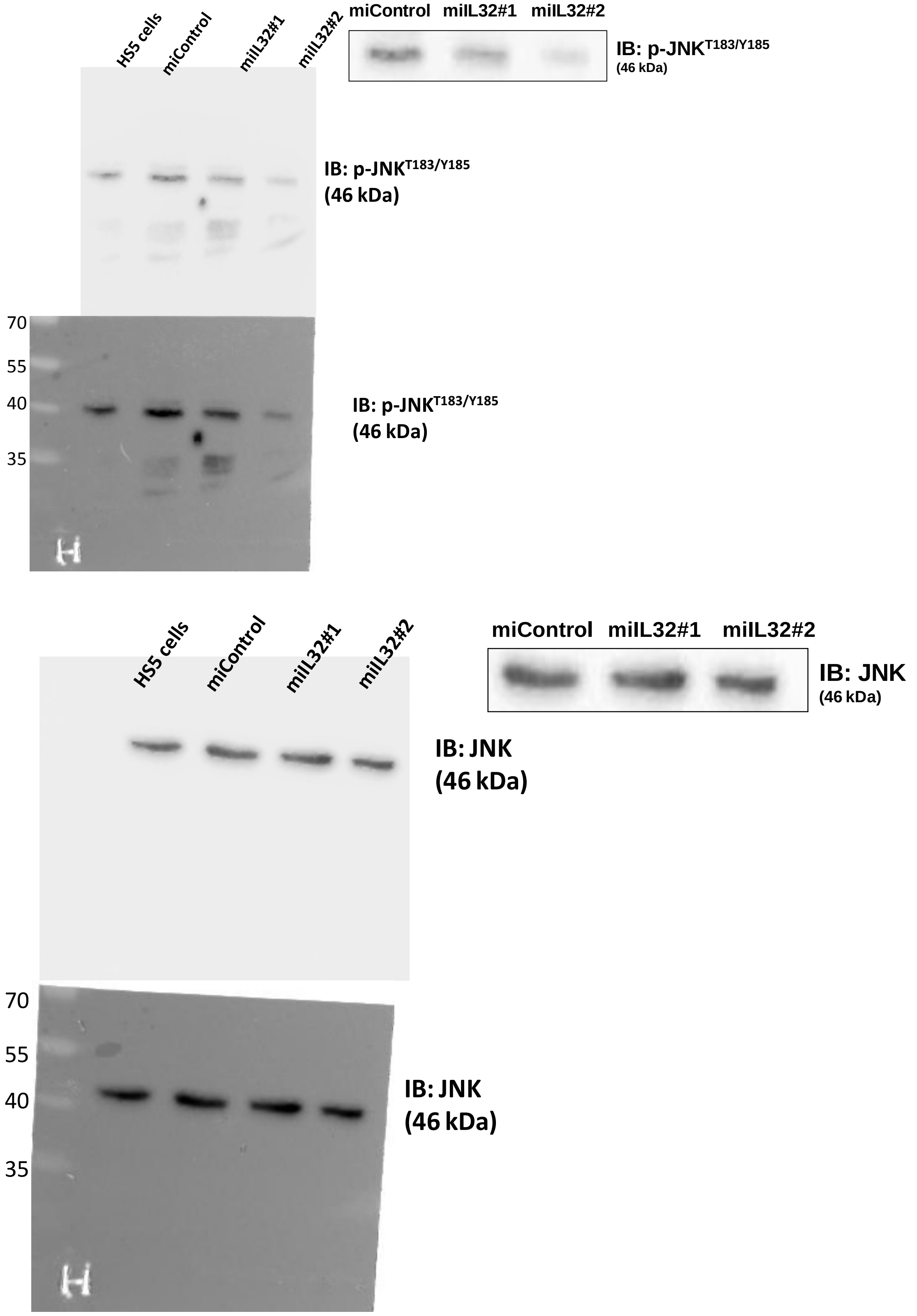

SUPPLEMENTARY FIGURE S3

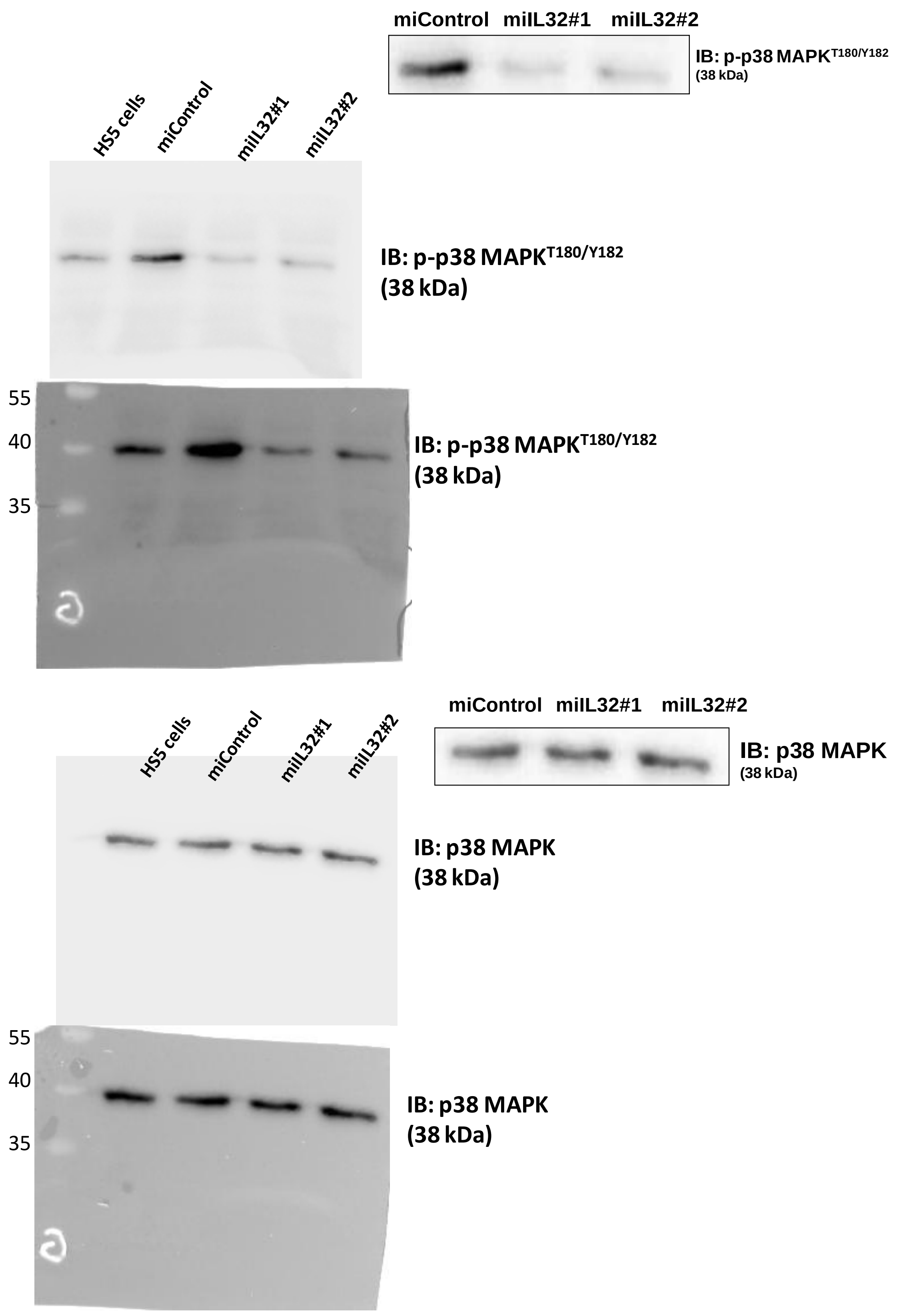

SUPPLEMENTARY FIGURE S3

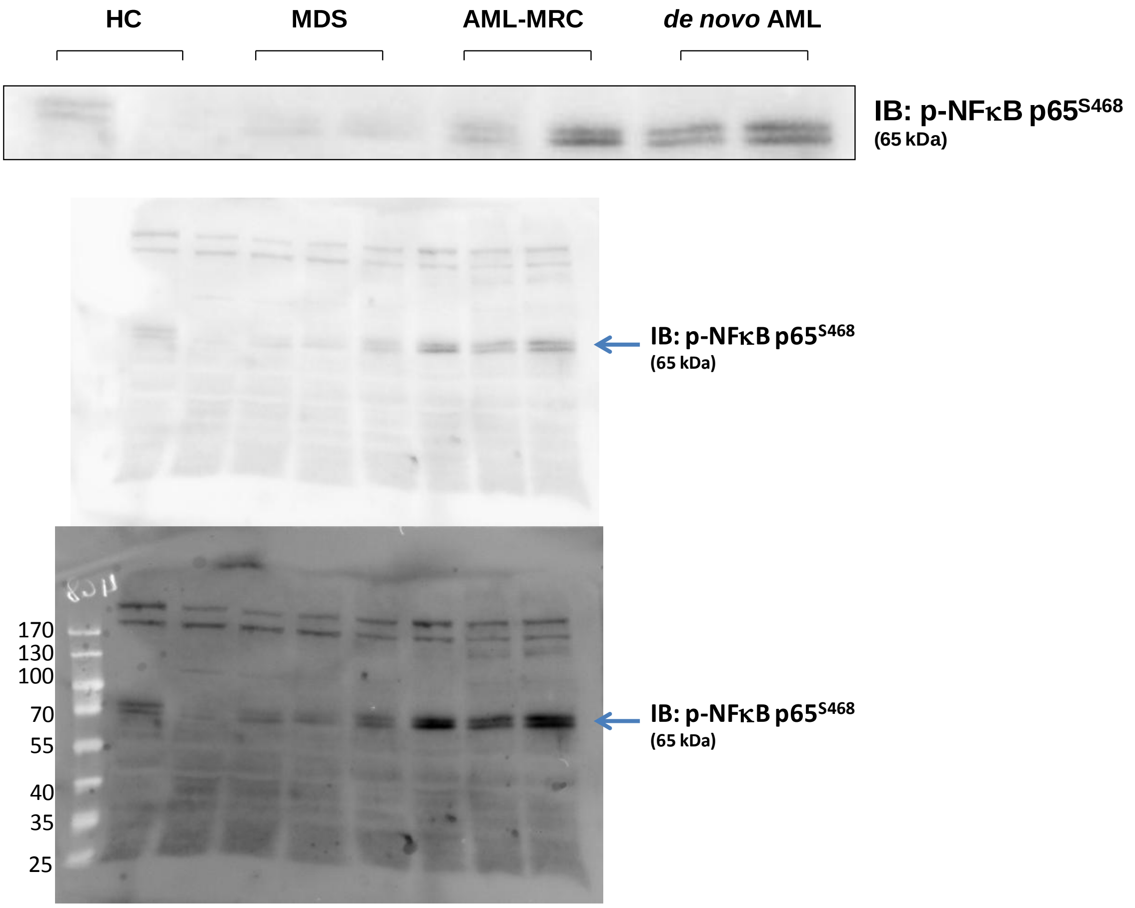

SUPPLEMENTARY FIGURE S3

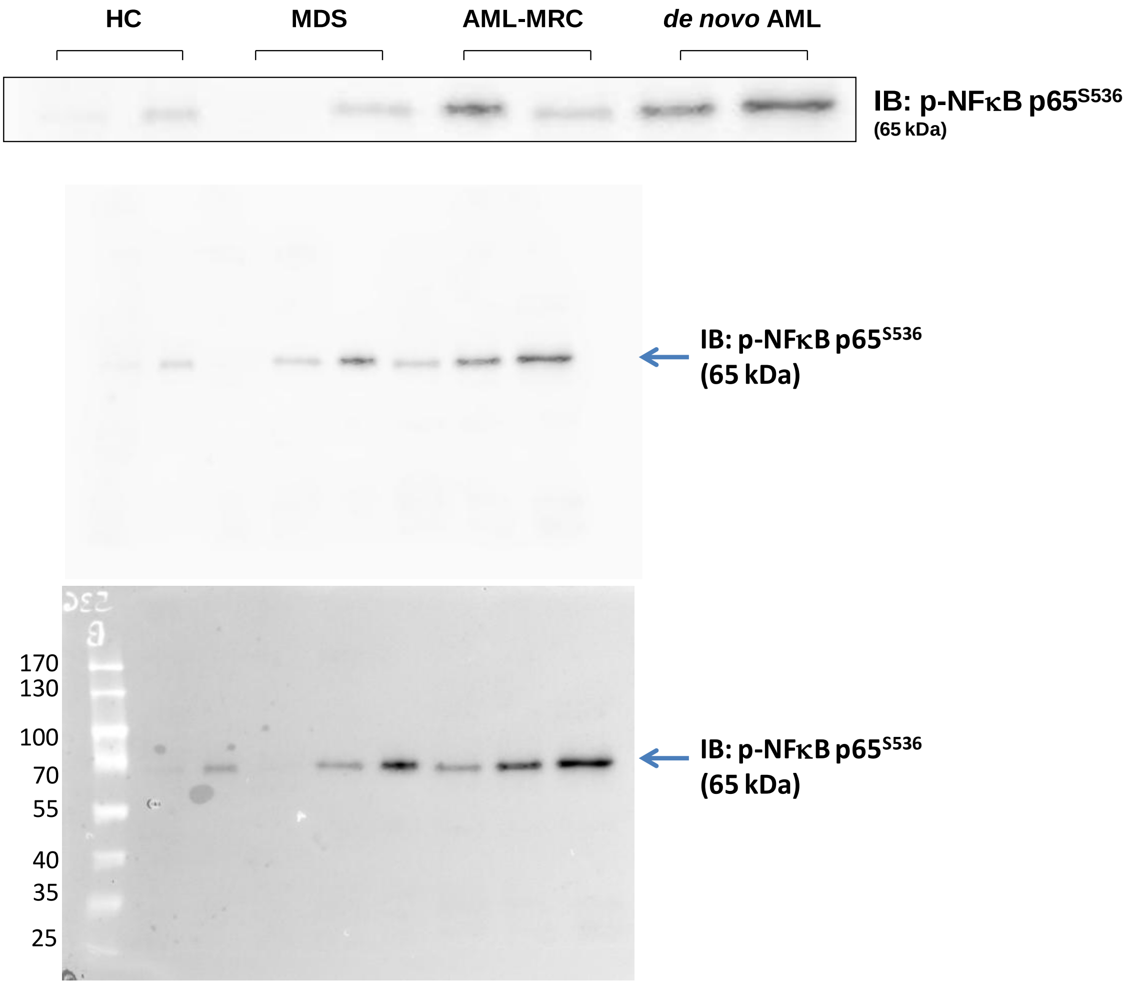

SUPPLEMENTARY FIGURE S3

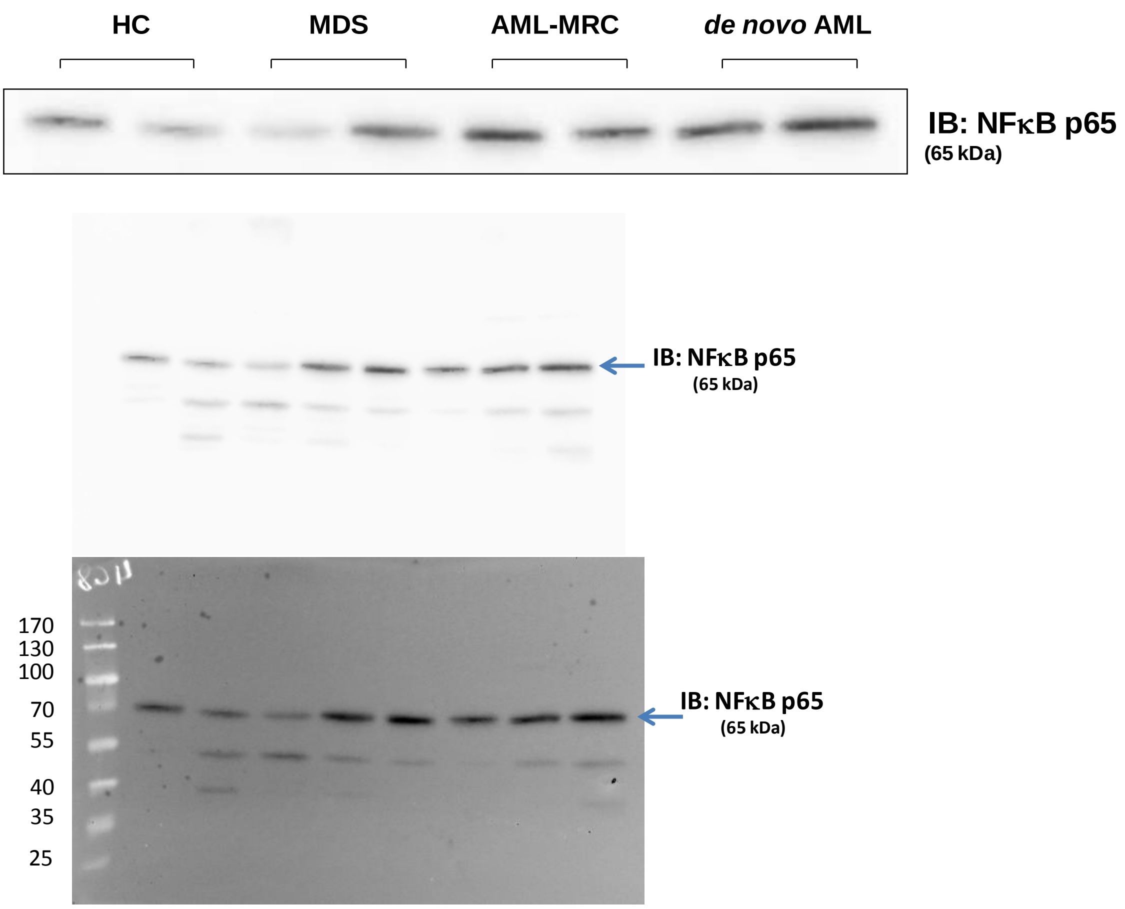

SUPPLEMENTARY FIGURE S3

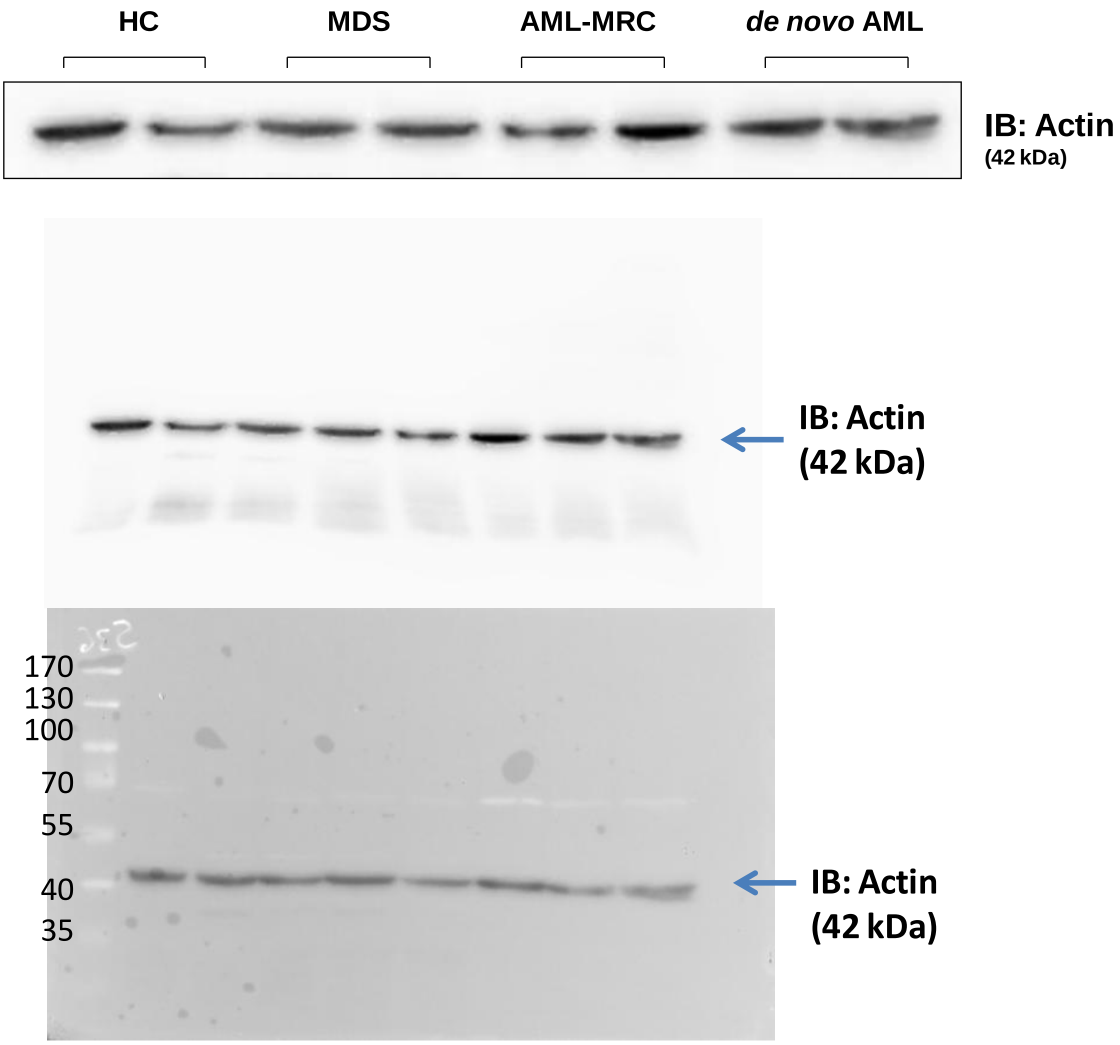

SUPPLEMENTARY FIGURE S3
